# Case Report: Synchronous primary malignancy including the breast and endometrium

**DOI:** 10.12688/f1000research.11971.3

**Published:** 2018-07-04

**Authors:** Elham Sadat Banimostafavi, Sepideh Tayebi, Maryam Tayebi, Fatemeh Montazer

**Affiliations:** 1 Radiology Department, Mazandaran University of Medical Sciences, Sari, Iran; 2 Medical School, Tehran University of Medical Sciences, Tehran, Iran; 3 Department of Pathology, Gastrointestinal Cancer Research Center, Imam Khomeini Hospital, Mazandaran university of Medical Sciences, Sari, Iran

**Keywords:** Breast, Endometrium, Cancer, Multiple Primary Malignancy

## Abstract

Breast and endometrial cancer are the most common types of female cancers, but the incidence of both of these malignancies in a single patient is a rare event. Multiple primary malignancy has been increasingly reported over the past decade, and double primary cancer is considered as the most common type.  In this study, we present a 53-year-old woman with synchronous primary malignancy of breast and endometrium. This patient had a history of breast and endometrial cancer in her family. Mammography and chest CT of the patient revealed a mass in the right breast and left supraclavicular region. However, the patient did not want to initiate treatment. Subsequently, the patient returned with a chief complaint of persistent abnormal vaginal bleeding. Abdominopelvic CT scan of the patient revealed a huge soft tissue mass in the pelvic cavity. She underwent hysterectomy, and pathology revealed endometrioid carcinoma, which had invaded the full thickness of uterine wall. Since this type of malignancy is rare and several risk factors are associated with it, it is worth being considered by clinicians when making decisions about screening or strategy for prevention.

## Introduction

Breast cancer (BC) is the most frequently diagnosed malignancy worldwide and is the first cause of cancer death in women ^1^. The common metastatic sites of breast cancer are the lungs, bones, liver and brain. Endometrial cancer (EC) is considered as the commonest type of gynecological cancer that mostly affecting post-menopausal women ^2^.

Multiple primary malignancy (MPM) has increased over the past decade. It is a term defined as occurring of the primary malignancy with different histology to two or more parts of the body distinct from each other. In addition to being distinct, these tumours must have definite featured of malignancy, and the possibility that one is the metastasis of the other must be ruled out ^3, 4^. Double primary cancers are the most common types of MPM.

Multiple mechanisms such as hereditary, immune and environmental factors, e.g. chemical, viruses and chemotherapeutic regimens, are considered as the pathogenesis of MPM ^5^. Tumours that are diagnosed simultaneously or within six months are known as synchronous; a longer interval time and the tumours are metachronous.

We present a patient with two primary malignant tumours, including BC (invasive ductal carcinoma) and EC (endometroid cell type), which can be considered as synchronous MPM.

## Case report

**Figure 1.**
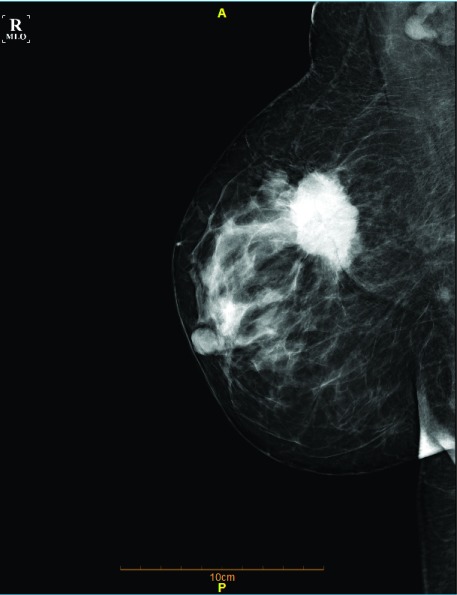
Breast mammography. An irregular speculated hyperdensity mass in the right breast upper outer quadrant.

**Figure 2.**
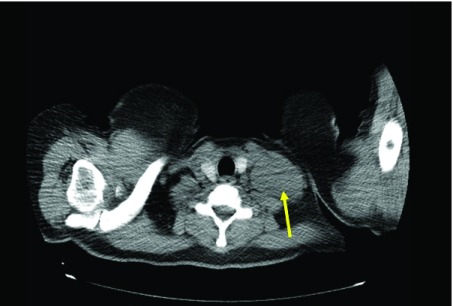
Chest CT scan. A soft tissue mass in the left supraclavicular region consistent with metastatic lymph node (yellow arrow).

The following case is a 53-year-old woman who was referred to hospital from a local doctor in December 2016 with a palpable mass in her left supraclavicular region. She was a post-menopausal woman with BMI of 29. Mammography and chest CT scan revealed no suspicious mass in the left breast, the presence of a speculated mass 3.8×3.7 cm in the right breast ( Figure 1), and additionally a soft tissue mass 5.8×5.1 cm in the left supraclavicular region ( Figure 2).

**Figure 3.**
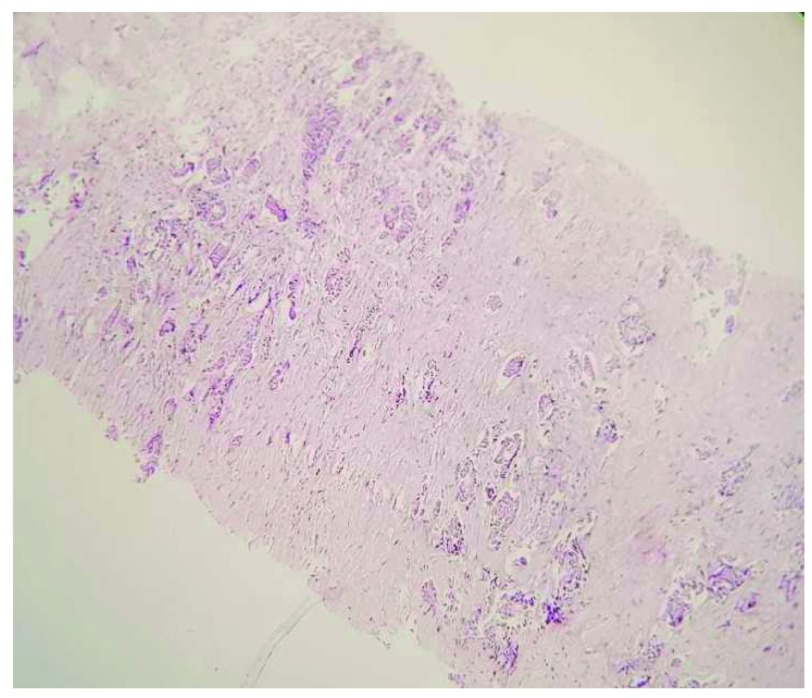
Breast invasive ductal carcinoma, CNB, H and E X100.

Core needle biopsy (CNB) for the right breast mass was preformed, and invasive ductal carcinoma (grade II) with involvement of axillary and supraclavicular lymph nodes was confirmed. On histopathology study, infiltrative cord and nest of neoplastic cells with moderate nuclear pleomorphism (score 2), scattered mitosis (score 1) and few tubular formation (score 3) were noted ( Figure 3).

Immunohistochemistry result for breast mass showed strongly positive staining for ER and PR in most tumor cells (3+5), 3+ staining for HER2new and 10% positive Ki67 in tumor cells. Although the mass was diagnosed as BC, the patient personally refused to get any treatment. She has a positive family history of breast cancer and uterine cancer in her sister.

**Figure 4.**
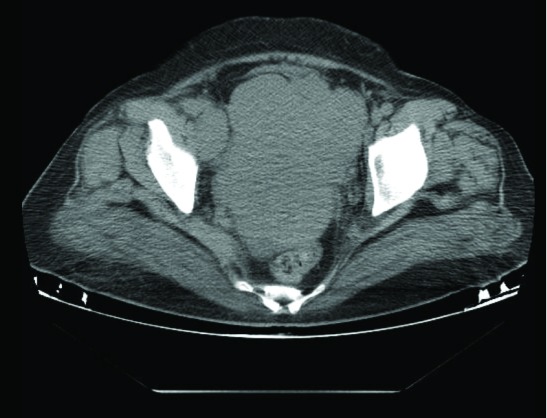
Abdominopelvic CT. A soft tissue mass in the pelvic cavity with right external iliac and para-aortic lymphadenopathy.

One month later, the patient returned with a chief complaint of persistent abnormal vaginal bleeding. She had the history of bleeding 4 years ago and it had worsened over the previous 7 months. Abdominopelvic CT scan of the patient revealed a huge soft tissue mass 14×11 cm in the pelvic cavity with right external iliac and para-aortic lymphadenopathy and dilatation of renal calyces and ureters on both sides ( Figure 4).

**Figure 5.**
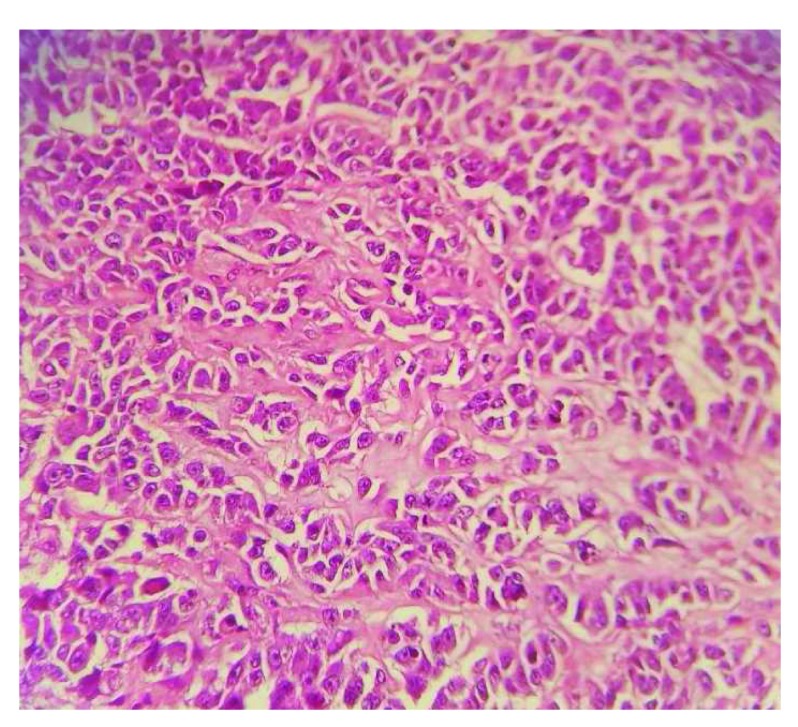
Endometrial carcinoma, H and E X400.

In January 2017, a total abdominal hysterectomy was performed with no complication, and the pathology revealed EC (stage IIIB, grade II). Pathology report showed sheets and cords of atypical cells with pleomorphism vesicular nuclei and visible nucleoli as well as frequent mitotic figure ( Figure 5). Extensive coagulative necrosis was also seen. Tumor cell had invaded the full thickness of the uterine wall. Pelvic wall mass resection and cervix excision revealed the invasion of the tumor, but peritoneal fluid cytology was negative for malignancy. No metastatic tumors have been found in this patient.

After two days she discharged from hospital with relative improvement. We could not follow up the patient because she moved to another city for further treatment; this is one limitation of our study. At the final follow-up, the patient was referred to the oncology department in a different hospital to initiate chemotherapy.

## Discussion

The diagnosis of synchronous primary cancers in an individual is rare and difficult ^6^. In the present case, clinicopathological criteria was used to distinguish the two similar cancers.

The risk of a new primary cancer in cancer survivors is 20% higher than in the general population ^7^. In addition, it has been shown that the risk of developing a new malignancy is 1.29 times more than those who have never been diagnosed ^8^. The possibility of synchronous BC and EC in one person is extremely low and might be only a coincidence, as reported in one study the diagnosis of EC within one year after the diagnosis of primary BC is less than 0.05%.

The coexistence of breast and endometrial cancer reflects the fact that there are many environmental and hormonal risk factors that may predispose the patient to both BC and EC, such as genetics, hormonal, environmental or treatment-related factors, and obesity (i.e. high BMI) ^9, 10^. Some of these factors are controversial. For instance, high BMI increases the risk of BC in postmenopausal women; however, it has opposite effect on premenopausal women ^11, 12^. By contrast, high BMI increases the risk of EC in both pre and postmenopausal women ^13, 14^.

There are many other situations that are correlated with an increasing risk of EC, such as age (i.e. more common in older patients), postmenstrual period ^15, 16^, nulliparous, and a positive history of irregular menstrual cycle ^13^. Our case had some of these risk factors, such as being postmenopausal and having a high BMI(=29).

Besides these factors, hormonal status has an important role in endometrial carcinogenesis. Lower exposure to estrogen and higher exposure to progesterone reduce the risk of EC ^17^. The conversion of adrenal hormones into estrogen may be done by fat cells in obese women, so obesity may increase the risk of EC in this way ^18^. Obesity, nullipara and irregular menstrual cycle may represent less progesterone exposure, so they may contribute to EC development. In addition, EC may develop in association with tamoxifen treatment for BC, particularly in the case of long-term administration and high cumulative doses of tamoxifen ^19– 21^. The patient in our study did not have any risk factors related to treatment because she did not start BC radio or chemotherapy before presentation of EC symptoms; therefore, we cannot consider the effects of tamoxifen usage in BC as a risk factor of EC in this patient.

Genetic and/or epigenetic changes and other plausible molecular mechanisms might be important in patients with synchronous double cancers ^22^. The present case had a family history of breast and uterine cancer, so heredity could be counted as one of the strongest risk factors for this patient.

In addition to many similar environmental and hormonal risk factors, the same embryological origin of the endometrium and breast can constitute as an additional factor ^5, 23^. MPMs can generally be categorized into three major groups depending on the main etiologic factor. The first group are treatment-related neoplasms, the second group are syndromic cases (like Cowden syndrome), and the third group are neoplasms that may share common etiologic factors, such as genetic predisposition or the same environmental factors ^24^. According to this classification, our patient can be categorized in the third group.

To conclude, finding a patient with simultaneous presentation of endometrial and breast cancer is rare; however both of these primary malignancies are considered as the most common cancers in females. Several associated risk factors to this event have been described above. In our case, a high BMI, postmenopausal status and hereditary are probably the most relevant risk factors. Hence, all these factors should be taken into account by clinicians when making a decision concerning screening or strategy for prevention.

## Consent

Written informed consent for the publication of the patient’s clinical details and images was obtained from the patient.
