# Supplementary material for: Case Report: Synchronous primary malignancy including the breast and endometrium
Source: F1000Res. 2018 Jul 4;6:1502. Originally published 2017 Aug 17. [Version 3] doi: 10.12688/f1000research.11971.3 (PMC5754747; doi:10.12688/f1000research.11971.3)
Supplement: Peer review history

# Peer review: Referee response for version 2

**DOI:** 10.5256/f1000research.14507.r29030

**Published:** 2018-01-04

**Authors:** Minas Sakellakis

**Affiliations:** 1 University of Texas MD Anderson Cancer Center, Houston, TX, USA

The diagnosis of synchronous breast and endometrial cancers might be challenging. The authors made an effort to address most of the the reviewers concerns. I think that the corrections now made the paper of acceptable quality and will be of value to clinicians.

I have read this submission. I believe that I have an appropriate level of expertise to confirm that it is of an acceptable scientific standard.

# Peer review: Referee response for version 1

**DOI:** 10.5256/f1000research.12943.r25974

**Published:** 2017-11-16

**Authors:** Minas Sakellakis

**Affiliations:** 1 University of Texas MD Anderson Cancer Center, Houston, TX, USA

This article needs to be significantly improved before it gets accepted for publication. Here are my suggestions to improve this article:

 COMMENTS ON INTRODUCTION:

- “Metastases to the gynecologic and gastrointestinal tract are rare ^2, 3^; endometrial cancer (EC) is considered as the most frequent of this type.”
  Please correct: Endometrial cancer is not a metastasis.
- “Multiple primary malignancy (MPM) has increased over the past decade. It is a term defined as spreading of the primary malignancy to two or more parts of the body distinct from each other. In addition to being distinct, these tumours must have definite featured of malignancy, and the possibility that one is the metastasis of the other must be ruled out”
  Please correct: Multiple primary malignancy is not spreading of the primary malignancy to two or more parts. The term refers to separate cancers.
- “Multiple mechanisms such as hereditary, immune and environmental factors, e.g. chemical, viruses and chemotherapeutic regimens, are considered as the pathogenesis of MPM”
  Many times (if not most times) MPM are the result of pure chance. For instance if a disease A has a prevalence of e.g. 3% and disease B has a prevalence of e.g. 2%, then a small number of patients will have both diseases only as a matter of chance. The same thing implies here for BC and EC.

 

 COMMENTS ON CASE PRESENTATION:

- Additional information should be given regarding the tumor stage. There is no information regarding the lymph node status in the right axilla. Does the text imply that the disease was spread directly to the left supra-clavicular lymph nodes. Did the authors rule out a second tumor in the left breast? Did they detect metastatic disease elsewhere?
- The authors don't mention the status of estrogen and progesterone receptors, nor do they report the HER2/NEU status.
- Pathology images should be added.

COMMENTS ON DISCUSSION:

- Language editing is strongly adviced before the authors submit a revision of this manuscript. For instance the phrase:"The diagnosis of synchronous primary cancers in an individual is rare and difficult, especially in the case of finding the same type of cancer. In the present case, clinicopathological criteria was used to distinguish the two similar cancer" needs to rephrased completely.
- Discuss that the coexistence of BC and EC in this patient might be only a coincidence.
- Information regarding menopausal status and BMI should be given in the case presentation section.
- What are the treatment implications?

I have read this submission. I believe that I have an appropriate level of expertise to confirm that it is of an acceptable scientific standard, however I have significant reservations, as outlined above.

# Peer review: Referee response for version 1

**DOI:** 10.5256/f1000research.12943.r26730

**Published:** 2017-10-12

**Authors:** Weibo Yu, Dylan Wan

**Affiliations:** 1 Department of Pathology and Laboratory Medicine, University of California, Los Angeles, Los Angeles, CA, USA

Breast cancer and endometrial cancer are two most frequent hormone-related cancers among women. The authors presented a rare case with synchronous primary malignancy of breast and endometrium. This is a well-written case report. It clearly described a particular individual’s history with a disease presentation and progress.

  

1. One limitation is the patient lost to follow up.
2. It is advised to add pathology images demonstrating their findings.
3. Patients with germline mutations in BRCA1 and BRCA2 genes are highly susceptible to breast cancer. These mutations are also related to an increased risk of developing endometrial cancer. Do author consider to perform an investigation in genetic level? It would be adequate to add more discussion in this point.

We have read this submission. We believe that we have an appropriate level of expertise to confirm that it is of an acceptable scientific standard.
